# Effect of pH on *in vitro* biocompatibility of orthodontic miniscrew implants

**DOI:** 10.1186/2196-1042-14-15

**Published:** 2013-07-01

**Authors:** Angela Galeotti, Roberto Uomo, Gianrico Spagnuolo, Sergio Paduano, Roberta Cimino, Rosa Valletta, Vincenzo D’Antò

**Affiliations:** Department of Pediatric Surgery, Bambino Gesù Children’s Hospital, Rome, Italy; Department of Neurosciences, Reproductive Sciences and Oral Sciences, University of Naples “Federico II”, Naples, Italy; Department of Clinical and Experimental Medicine, University of Catanzaro “Magna Graecia”, Catanzaro, Italy

**Keywords:** Biocompatibility, Miniscrew implants, TADs, Titanium alloy, Stainless steel

## Abstract

**Background:**

Although the clinical use of miniscrews has been investigated on a large scale, little is known about their biocompatibility. Since low pH can affect corrosion resistance, the aim of this study was to evaluate the cytotoxic effect of orthodontic miniscrews in different pH conditions.

**Methods:**

Four orthodontic miniscrews of stainless steel and grade IV and grade V titanium were immersed in a pH 7 and pH 4 saline solution for 1, 7, 14, 21, 28, and 84 days. Human osteogenic sarcoma cells (U2OS), permanent human keratinocytes (HaCat), and primary human gingival fibroblasts (HGF) were exposed to eluates, and the mitochondrial dehydrogenase activity was measured after 24 h to assess the cytoxicity. The results were analyzed using the Mann-Whitney *U* test (*P* < 0.05).

**Results:**

When exposed to pH 7-conditioned eluates, the cell lines showed an even greater viability than untreated cells. On the contrary, the results revealed a statistically significant decrease in U2OS, HaCat, and HGF viability after exposure to eluates obtained at pH 4. Among the cell lines tested, HGF showed the most significant decrease of mitochondrial activity. Interestingly, grade V titanium miniscrews caused highest toxic effects when immersed at pH 4.

**Conclusions:**

The results suggested that at pH 7, all the miniscrews are biocompatible while the eluates obtained at pH 4 showed significant cytotoxicity response. Moreover, different cell lines can produce different responses to miniscrew eluates.

## Background

Orthodontic temporary anchorage devices (TADs) are successfully used as compliance-independent anchorage devices in orthodontics to prevent many of the shortcomings of traditional anchorage methods [1]. Removable miniscrews are available in a range of body lengths and diameter, and are mainly made of pure titanium, titanium alloy, and stainless steel (SS) [2]. Literature in this regard has reported a great number of clinical studies suggesting that TADs may provide stable anchorage during the orthodontic treatment without requiring patient cooperation [3].

In an oral environment, miniscrews are exposed to a number of potentially damaging physical and chemical agents. Such conditions may contribute to corrosion of the metal components of any intraoral appliance, and an increasing number of studies have demonstrated that the oral cavity, owing to its peculiar physical, chemical, enzymatic, and microbial characteristics, may play a significant role in the biodegradation of metallic biomaterials [4, 5]. Food intake and microbial flora can induce a decrease in the physiologic pH of saliva (5.3 to 7.8) [6, 7]. Although buffering action is an important function of saliva (mainly through bicarbonate, phosphate, urea) [7], these transient variations may affect corrosion resistance of metal devices [8]. Many studies have investigated the role of pH in metal ion release from orthodontic alloys. The majority of them, in different experimental conditions, report ion release increase on decrease of the pH [9–15].

Moreover, although the systemic level of metallic ions may not reach toxic concentration [16], the degradation products may be a potential source of local adverse biologic effects, such as toxicity, mutagenicity, and allergenicity [17–20].

Cytotoxicity tests are used to estimate the biologic safety of medical devices. *In vitro* experiments, assessing cytotoxicity of orthodontic appliances, have also been performed. Previous studies have reported a good biocompatibility of metallic brackets and archwires [21–23]. Mild toxicity was found by Oh and Kim when testing SS wire extracts on fibroblasts [24]. Kao et al. found that both SS and nickel-titanium wires can cause cell toxicity when immersed in acidic saliva solution [14].

Literature in this matter lists a wide array of articles dealing with orthodontic miniscrews; however, the majority of them is limited to case reports, preliminary reports, or case series studies, and the information provided is lacking [25].

To our knowledge, there is no study which has tested the cytotoxicity of miniscrews; they are considered biocompatible because they are made of medical stainless steel and type IV or type V titanium alloy. Recent reviews underline that knowledge on miniscrew biocompatibility is mainly grounded on the cytotoxic effects of the alloy used [25].

Since orthodontic miniscrews are fixed to the bone, through soft tissues, it is essential to determine the relative levels of biocompatibility of the various mini-implants. As such information is not available, the aim of this work was to study the cytotoxic effects on primary human oral gingival fibroblasts (HGFs), a permanent human osteogenic sarcoma cell line (U2OS), and a permanent human keratinocyte cell line (HaCaT) of four orthodontic mini-implants made of different alloys (surgical stainless steel, grade IV and grade V titanium alloy) which were stored under different pH conditions.

## Methods

### Sample preparation

Four different miniscrews for orthodontic anchorage were investigated (Table 1): Miniscrew Anchorage System® (MAS, Micerium S.p.a., Avegno, Italy), Orthodontic Mini-Implants® (OMI, Leone S.p.a., Florence, Italy), Spider Screw anchorage system® (HDC S.r.l., Sarcedo, Italy), and Ortho Screw® (Novaxa S.r.l., Cinisello Balsamo, Italy).

**Table 1:** Details of miniscrew implant system tested in this study

Miniscrew	Company	Alloy	Order no.
Orthodontic mini-implants® (OMI)	Leone S.p.a., Firenze, Italy	Surgical stainless steel AISI 316	000-2012-02
Spider screw anchorage system® (SSAS)	HDC S.r.l., Sarcedo, Italy	Grade IV titanium	SSM-2009
Miniscrew anchorage system® (MAS)	Micerium S.p.a., Avegno, Italy	Grade V titanium	AM1311
Ortho screw® (OS)	Novaxa S.r.l., Cinisello Balsamo, Italy	Grade V titanium	IO1607

Sterile samples were immersed in 0.9% (*w/v*) NaCl solution in airtight test tubes (Sterilin, Barloworld scientific, Staffordshire, UK). The pH of immersion media was adjusted to 7 and 4 using NaOH and HCl. Samples were stored under stationary conditions at 37°C, and after 1, 7, 14, 21, 28, and 84 days, the solutions were removed and fully replaced with fresh sterile saline solution. The eluates and the saline solutions stored under the same conditions were diluted in culture medium (25%, *v/v*) and used for the cytotoxicity test. The ratio between the volume of the final dilution and the weight of the samples was 1 ml/0.1 mg, as recommended by the International Standards Organization [26].

### Cell cultures

All cell culture media and reagents were obtained from Sigma Chemical Company (St Louis, MO, USA). Primary human gingival fibroblasts (HGF) were cultured using an explant technique. Gingival tissue was obtained from surgical operations (e.g. frenulectomies, flap operations) in healthy 20- to 30-year-old patients, under informed consent approved by the Institutional Review Board (University of Napoli “Federico II”). Only the connective layer was dissected from the gingival samples by means of a surgical blade. Tissue fragments were washed twice in phosphate buffered saline (PBS) and transferred into tissue culture dishes in Dulbecco's minimal essential medium supplemented with 10% fetal bovine serum (FBS), 2 mM glutamine, 100 U/ml of penicillin and 100 mg/ml of streptomycin at 37°C in a humidified atmosphere of 5% CO_2_ in air. After 10 days, fragments were removed, and the released fibroblasts started proliferating. Once the confluence was reached, cells were washed with PBS and detached from the culture dishes using a brief treatment with trypsin/EDTA for 5 min and recultured until confluent monolayer was again obtained. In all the experiments, cells were used between passage 4 and passage 6.

Immortalized human keratinocytes (HaCaT) were grown in the same conditions. Only early passages (<50) were used for the experiments. Human osteogenic sarcoma cells (U2OS) were grown in McCoy's medium supplemented with 10% FBS, 2 mM glutamine, 100 U/ml of penicillin, and 100 mg/ml of streptomycin at 37°C in a humidified atmosphere of 5% CO_2_ in air. Confluent cells were detached and recultured as above mentioned.

### Evaluation of cell viability

[3-(4,5-Dimethylthiazol-2-yl)-2,5-diphenyl tetrazolium bromide] (MTT) assay was used to evaluate cell viability, measuring mitochondrial dehydrogenase activity as previously described [27]. HaCat, HGF, and U2OS cells were plated into 96-well flat bottom, tissue culture plates, respectively, at 10^4^ cells/well. After 24 h of incubation, the culture medium was replaced with 200 μl/well of dilutions. After further 24 h, the medium was then replaced with 100 μL/well of MTT solution (1 mg/ml) in PBS, and the cells were incubated for an additional h at 37°C in a 5% CO_2_ atmosphere. After the solution was removed, 100 μL/well of DMSO were added, and the plates were swirled gently for 10 min. The optical density of each well was immediately measured in a spectrophotometer (Sunrise™, Tecan, Mannedorf/Zurich, Switzerland) at 590 nm.

The optical density of cells cultured in the medium plus saline solution without the miniscrew extracts was used as control for 100% cell viability and as reference for the determination of the cytotoxicity (%) in the assay. At least four independent experiments were performed in quadruplicate.

Since cell metabolism and growth vary in cell line cultures, we included nickel chloride (5 mM) in each assay to check for possible cell susceptibility variation. The 5-mM NiCl_2_ solution, our positive control, showed a severe cytotoxicity, and no significant variations were found between the experiments (data not shown).

### Statistical analysis

The differences in median values were statistically analyzed using the Mann-Whitney *U* test for pairwise comparisons among groups at the 0.05 level of significance.

## Results

The morphology of the cells appeared to have been maintained subsequent to mini-implant immersion media exposure. Cell membranes appeared to be intact without damage or apoptosis (data not shown).

### Effect on HaCat cells

There was a statistically significant difference on HaCat cells among eluates obtained after immersion of stainless steel miniscrew (OMI). In particular, after 7 days of immersion at pH 4, there was a significant decrease in HaCat viability. A slight increase in metabolic activity was observed after 21 days in both pH-conditioned eluates.

Grade IV titanium miniscrew (SSAS) eluates demonstrated the least influence on mitochondrial activity on HaCat cells: only samples obtained after 21 days of immersion at pH 7 were statistically different from the control showing higher cell viability value.

The two tested grade V titanium miniscrews caused different effects on HaCat cells. Mitochondrial activity values had significant increases at pH 7 after 7 days and after 21 days of MAS immersion; at pH 4 a significant increase in cell viability was observed after 21 days.

**Figure 1:**
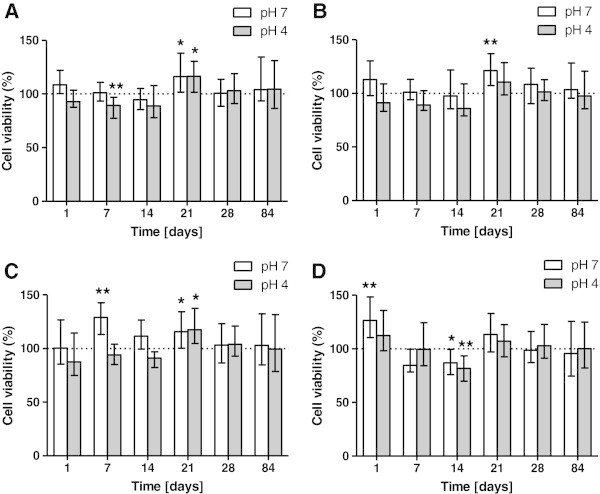
**Survival rate comparison of HaCat cells in miniscrew immersion media.** According to immersion time solution at two different pH: Leone OMI **(A)**, HDC SSAS **(B)**, Micerium MAS **(C)**, Novaxa OM **(D)**. The bars represent medians (25% and 75% percentiles) calculated from 16 individual values (*n* = 16) in four independent experiments. (Asterisk) and (double asterisk) indicate significant differences between treated and untreated cell cultures respectively at *P* < 0.05 and *P* < 0.01.

OS eluates caused a significant increase in cell viability after 1 day of immersion at pH 7. Moreover, a significant decrease in mitochondrial activity was observed after 14 days of immersion both at pH 7 and 4 (Figure 1).

### Effect on HGF cells

Stainless steel miniscrew (OMI) eluates did not lead to significant alteration in HGF metabolism except for the 1-day extract, when a slight increase was observed at pH 7. On the other hand, at pH 4, a significant decrease was found after 7 and 14 days.

SSAS extracts caused decreases in HGF viability. After 7 days of immersion at pH 7, a significant reduction in mitochondrial activity was seen. On the other hand, at pH 4, significant decreases were found when HGFs were exposed to eluates obtained after 1, 7, 14, 21, 28, and 84 days. MAS showed a cytotoxic response after 7 days of immersion at both pH 7 and pH 4 and after 14 days of immersion. A significant decrease in cell viability was also shown after HGFs' exposition to pH 4 eluates after 21, 28, and 84 days.

**Figure 2:**
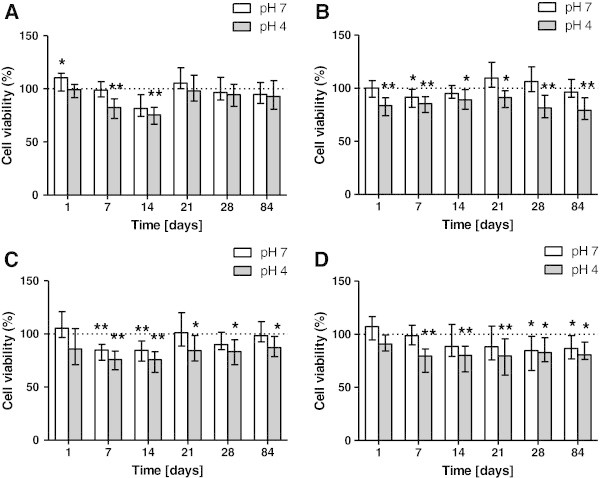
**Survival rate comparison of HGF cells in miniscrew immersion media.** According to immersion time solution at two different pH: Leone OMI **(A)**, HDC SSAS **(B)**, Micerium MAS **(C)**, Novaxa OM **(D)**. The bars represent medians (25% and 75% percentiles) calculated from 16 individual values (*n* = 16) in four independent experiments. (Asterisk) and (double asterisk) indicate significant differences between treated and untreated cell cultures respectively at *P* < 0.05 and *P* < 0.01.

OS extracts at pH 4 caused significant viability reduction after 7, 14, 21, 28, and 84 days. pH 7 eluates resulted slightly cytotoxic after 28 and 84 days of immersion periods (Figure 2).

### Effect on U2OS cells

OMI eluates significantly reduced cell viability with respect to the control cells after 1 and 7 days of immersion at pH 4 and after 7 and 14 days of immersion at pH 7.

MAS eluates were citotoxic after 1 day of immersion, causing the lowest survival value on U2OS cells (64.7 ± 15.8% survival rate) at pH 4. A slight decrease was observed at pH 7 after 1-day exposure, while after 7 and 14 days of immersion at pH 4.

**Figure 3:**
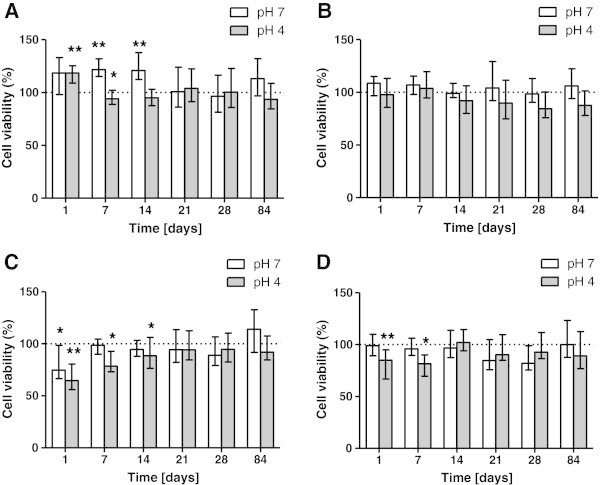
**Survival rate comparison of U2OS cells in miniscrew immersion media.** According to immersion time solution at two different pH: Leone OMI **(A)**, HDC SSAS **(B)**, Micerium MAS **(C)**, Novaxa OM **(D)**. The bars represent medians (25% and 75% percentiles) calculated from 16 individual values (*n* = 16) in four independent experiments. (Asterisk) and (double asterisk) indicate significant differences between treated and untreated cell cultures respectively at *P* < 0.05 and *P* < 0.01.

OS resulted to have the least effects on U2OS metabolism. A slight decrease in mitochondrial activity was observed at pH 4, after low time exposure, 1 and 7 days (Figure 3).

## Discussion

Previous studies have shown that different components of orthodontic appliances, such as bands, brackets and archwires may release metal ions *in vivo*[5] and under *in vitro* conditions [28]. The release of ions from orthodontic materials is of particular importance due to the possibility of cytotoxic, mutagenic, and immunotoxic effects [17–20, 29]. Furthermore, many papers reported on dental implant failures due to multiple reasons. Therefore, the question as to whether implant materials contribute to implant failures or whether adverse effects of dental implant materials exist at all is somewhat difficult to answer [30].

In this study, we tested the biocompatibility of miniscrew implants made of different alloys. Cytotoxic effects have been evaluated on different cell lines after immersion in saline solution at pH 4 and pH 7, for 1, 7, 14, 21, 28 and 84 days. The immersion in a solution at pH 4 up to 84 days is not a true condition in the oral environment, but it was chosen to simulate the clinical situation of soft tissues inflammation around the miniscrew implants.

The specimens were stored in 0.9% NaCl, which is higher salinity than in saliva [31]. *In vitro* studies using artificial saliva may give a more realistic picture of nickel and chromium release. However, we decided to use 0.9% sodium chloride, rather than an artificial saliva as a test solution, because its use has been widely validated in previous studies [32–36], and it is specified in ISO protocol [26]. Moreover, earlier reports indicate that *in vitro* corrosion studies with physiologic saline solutions produced results comparable with those conducted in blood and other extracellular fluids, and similar results may be found in saliva [37–39]. In particular, under our experimental conditions, the immersion media was diluted, and the final concentration was 25% (*v/v*) to avoid further culture medium dilution and thus adverse effects on cell physiology.

Cellular metabolic activity was assessed by means of the MTT assay in order to evaluate the activity of mitochondrial succinic dehydrogenase by measuring the amount of formazan produced by this enzyme [27]. This method is widely used to test the biocompatibility of orthodontic materials [40, 41] and was chosen to evaluate the relative toxicity to tested cells of orthodontic miniscrew implant extracts. The use of the MTT test was justified for two main reasons: the measurements are fast and easy to carry out and, more importantly, the results can be compared with similar investigations.

Under our experimental conditions, the eluates obtained at pH 7 were scarcely cytotoxic. Moreover, in agreement with previous studies [42], after exposure to miniscrew eluates, the cells showed a mitochondrial activity even greater than untreated controls, suggesting a proliferative response to a small amount of released ions.

The clinical relevance of the *in vitro* cytotoxicity studies has been widely debated [43]. In general, the lack of *in vitro* cytotoxicity of the tested materials corresponds to the good clinical history of these materials and shows a good correlation between the lack of *in vitro* cytotoxicity and the lack of clinical problems. The correlation is more difficult to establish in the case of a positive response, i.e., when the materials are cytotoxic *in vitro*, but it also is not possible to eliminate *in vitro* and animal tests because we cannot use clinical tests to evaluate novel materials - legally, ethically, or scientifically [43].

Here, the eluates obtained at pH 4 showed a decrease of cell viability and that cytotoxicity was significantly dependent on the cell line selected for the test. Our results are in agreement with those of previous investigators who have reported that different cell lines respond to biomaterials differently [44, 45]. The decrease of cell metabolic activity was probably caused by the release of metal ions from the miniscrew tested. This result was in line with other studies that have revealed that a greater number of metal ions are released in solution from orthodontic appliances immersed in acid solutions [9–15].

In our experimental setting, miniscrew implants resulted to have the least effects on U2OS metabolism. A decrease in mitochondrial activity could be observed due to exposure to eluates obtained after 1 day and 7 days, but it was not confirmed at later time points.

Stainless steel miniscrews were at the same level of biocompatibility of titanium mini-implants. The different chemical compositions of the alloys not only characterize the physical and mechanical properties, of great importance in the biomechanics of the tooth movement, but also affect the biological properties, which are of even greater importance in terms of biocompatibility. Austenitic steels that contain Ni as the primary austenite stabilizer include the 316 L type that is the stainless steel most commonly used for implantation applications. Some concern has been expressed on the corrosion potential and Ni release of the AISI type 316 L austenitic stainless steel alloy currently used in orthodontics [46]. Because the Ni atoms are not strongly bonded to form some intermetallic compound, the likelihood of *in vivo* slow Ni ion release from the alloy surface is increased, which may have implications for the biocompatibility of these alloys [39, 47]. Titanium is the most commonly selected material for dental implants, due to its mechanical properties, good resistance to corrosion in biological fluids, and very low toxicity [48–51]. The stability of titanium under corrosion conditions is essentially due to the formation of a stable and tightly adherent thin protective oxide layer on its surface [52, 53]. The passive film stability depends on its structure and composition, which in turn are dependent on the conditions in which it was formed [52, 53]. pH is known to have a strong influence on the corrosion resistance of Ti and Ti alloys [54]. Ion release to the surroundings takes place when the dissolution of the surface passive film is accompanied by corrosion of the underlying base material. Therefore, release of ions from titanium implants may result in adverse biological reactions [51, 53].

## Conclusion

This *in vitro* study showed that the four types of miniscrews appear to be biocompatible with HaCat, HGF, and U2OS cells. However, our results showed a decrease of cell viability caused by eluates obtained at pH 4, suggesting the importance of oral environment in the biocompatibility of dental materials. The stainless steel miniscrews showed the same response of titanium mini-implants, although the presence of nickel might be a concern for patients with nickel hypersensitivity.
